# Evidence for Dose-Additive Effects of Pyrethroids on Motor Activity in Rats

**DOI:** 10.1289/ehp.0900667

**Published:** 2009-06-08

**Authors:** Marcelo J. Wolansky, Chris Gennings, Michael J. DeVito, Kevin M. Crofton

**Affiliations:** 1 Departamento de Química Biológica (Área Toxicología), Facultad de Ciencias Exactas y Naturales, Universidad de Buenos Aires, Ciudad Universitaria, Buenos Aires, Argentina; 2 Solveritas, LLC, Richmond, Virginia, USA;; 3 Division of Experimental Toxicology and; 4 Division of Neurotoxicology, National Health and Environmental Effects Research Laboratory, Office of Research and Development, U.S. Environmental Protection Agency, Research Triangle Park, North Carolina, USA

**Keywords:** additivity, cumulative, mixtures, neurotoxicity, pyrethroids

## Abstract

**Background:**

Pyrethroids are neurotoxic insecticides used in a variety of indoor and outdoor applications. Previous research characterized the acute dose–effect functions for 11 pyrethroids administered orally in corn oil (1 mL/kg) based on assessment of motor activity.

**Objectives:**

We used a mixture of these 11 pyrethroids and the same testing paradigm used in single-compound assays to test the hypothesis that cumulative neurotoxic effects of pyrethroid mixtures can be predicted using the default dose–addition theory.

**Methods:**

Mixing ratios of the 11 pyrethroids in the tested mixture were based on the ED30 (effective dose that produces a 30% decrease in response) of the individual chemical (i.e., the mixture comprised equipotent amounts of each pyrethroid). The highest concentration of each individual chemical in the mixture was less than the threshold for inducing behavioral effects. Adult male rats received acute oral exposure to corn oil (control) or dilutions of the stock mixture solution. The mixture of 11 pyrethroids was administered either simultaneously (2 hr before testing) or after a sequence based on times of peak effect for the individual chemicals (4, 2, and 1 hr before testing). A threshold additivity model was fit to the single-chemical data to predict the theoretical dose–effect relationship for the mixture under the assumption of dose additivity.

**Results:**

When subthreshold doses of individual chemicals were combined in the mixtures, we found significant dose-related decreases in motor activity. Further, we found no departure from the predicted dose-additive curve regardless of the mixture dosing protocol used.

**Conclusion:**

In this article we present the first *in vivo* evidence on pyrethroid cumulative effects supporting the default assumption of dose addition.

Pyrethroids are synthetic insecticides derived from pyrethrins (Casida 1980; Elliott 1978). Pyrethroids are increasingly used in a wide array of pesticide applications, including veterinary, agriculture, and home pest control (Amweg et al. 2005). Recent reports indicate low-level exposure to multiple pyrethroids in humans (Becker et al. 2006; Fortin et al. 2008; Heudorf et al. 2004; Lu et al. 2006, 2009; Morgan et al. 2007; Riederer et al. 2008; Tulve et al. 2006). Pyrethroids have been classified as type I or type II based on acute high-dose biological effects and chemical structure (Gammon et al. 1981; Gray 1985; Lawrence and Casida 1982; Verschoyle and Aldridge 1972, 1980). Type I compounds lack an α-cyano group on the phenoxybenzyl moiety and produce toxic signs characterized by aggressive sparring and tremors (T syndrome). Type II compounds contain an α-cyano group on the phenoxybenzyl moiety, and acute exposures produce a syndrome characterized by choreoathetosis and salivation (CS syndrome). A limited number of pyrethroids elicit both tremors and salivation (Gammon et al. 1981; Lawrence and Casida 1982; Verschoyle and Aldridge 1980) and have been classified as type I/II or TS syndrome compounds.

The Food Quality Protection Act (FQPA 1996) requires the U.S. Environmental Protection Agency (EPA) to consider the cumulative risk of chemicals having a common “mechanism of toxicity.” For chemicals considered to have a common mechanism of toxicity (commonality of target tissue, target site, and primary toxicologic effects for the members of a chemical class), dose additivity is the default hypothesis for assessing the hazard of mixtures ([Bibr b70-ehp-117-1563][Bibr b72-ehp-117-1563]). The overall assumption under dose additivity is that the toxicity of each component of the mixture behaves as a known dilution of a reference chemical selected as the index compound (U.S. EPA 2000). This approach was used for the cumulative risk assessment of cholinesterase-inhibiting organophosphate (U.S. EPA 2002b) and carbamate (U.S. EPA 2007) pesticides. The present study was part of a larger research effort to determine whether or not pyrethroid pesticides share a common mechanism and may thus be subject to cumulative risk assessment.

Pyrethroids act primarily on the nervous system (Narahashi 2000; Soderlund et al. 2002). A proposed mechanism of action for all pyrethroids is the prolongation of the open state of neuronal voltage-dependent sodium channels (Narahashi 1971; Vijverberg and van den Bercken 1990). This action results in altered neuronal excitability characterized by *in vitro* and *in vivo* changes in neuronal firing rates (e.g., repetitive firing or depolarizing block of the neuron) (Narahashi 2000) that are associated with two high-dose neurologic syndromes, T syndrome and the CS syndrome (Aldridge 1990; Barnes and Verschoyle 1974; Lawrence and Casida 1982; Ray and Forshaw 2000; Ray and Fry 2006; Verschoyle and Aldridge 1980), and dose-dependent changes in motor and sensory behaviors at lower doses (Chanh et al. 1984; Crofton and Reiter 1984 1988; Hornychova et al. 1995; McDaniel and Moser 1993; Nishimura et al. 1984; Wolansky et al. 2006; Wolansky and Harrill 2008).

Currently, the link between alterations in neuronal firing and downstream neurobehavioral syndromes is correlative and not causative. Mechanistic pathways linking sites of action (e.g., sodium channels) to neurologic outcomes have not been fully elucidated (Gray 1985; Ray and Fry 2006; Shafer et al. 2005; Soderlund et al. 2002). This has resulted in uncertainty about whether a common mechanism of toxicity exists for all pyrethroids (Soderlund et al. 2002). Pyrethroid actions on many other neuronal target sites have been reported and include calcium (Ca^++^), potassium (K^+^), and chloride (Cl^−^) channels (Burr and Ray 2004; Lawrence and Casida 1982; Ray and Fry 2006; Shafer and Meyer 2004). Although those mechanisms of action are not as well established (Shafer and Meyer 2004) as actions on Na^+^ channels, alterations in these ion channels will also disrupt neuronal firing rates.

No published data exist to determine whether dose addition predicts the effects of the combined exposure to pyrethroids in mammals. Some *in vitro* evidence on the action of pyrethroid mixtures has been reported. Whole-cell and patch-clamp assays in cultured neurons from rat suggest that type I and type II pyrethroids interact with Na^+^ channel binding sites by either competitive or allosteric actions (Motomura and Narahashi 2001; Song and Narahashi 1996). In addition, structure-dependent interaction among pyrethroids has been proposed to occur in Cl^−^ channels: pretreatment with *cis*-resmethrin (type I) antagonizes the effects of fenpropathrin, a mixed type I/II pyrethroid (Burr and Ray 2004). Unfortunately, these studies were not designed to test the hypothesis of dose addition (Burr and Ray 2004). Soderlund et al. (2002) highlighted the need for empirical data to test the hypothesis of additivity for pyrethroids using robust statistical models (e.g., Casey et al. 2004; Feron and Groten 2002; Olmstead and LeBlanc 2005).

In the present study, we tested the hypothesis that pyrethroids act in a dose-additive manner. The hypothesis was tested using the “single-chemical-required” (SCR) method (Casey et al. 2004) that compares a threshold additivity model (Gennings et al. 2004), fit to single-compound data, with a similarly parameterized model for data obtained from an experimentally tested mixture. We used motor activity as the dependent variable in this study for two reasons. First, motor activity is a valid test method routinely used in acute and subchronic regulatory neurotoxicity studies [Organization for European Cooperation and Development (OECD) 1997, 2007; Soderlund 2001; Soderlund et al. 2002; U.S. EPA 1998a, 1998b). Second, motor activity has been extensively evaluated for a number of pyrethroids in rodents. Seventeen pyrethroid preparations, assessed under a variety of dosing and testing conditions across laboratories, produced decreased activity (Crofton et al. 1995; Crofton and Reiter 1984, 1988; De Souza Spinosa et al. 1999; Hornychova et al. 1995; Hoy et al. 2000; Mandhane and Chopde 1997; McDaniel and Moser 1993; Reiter et al. 1981; Righi and Palermo-Neto 2003; Wolansky et al. 2006). In addition, recent research has characterized extensive dose–effect functions for 11 pyrethroids on motor activity. Data from these 11 compounds were used to compute ED_30_ values (dose that decreases activity by 30%) and relative potencies (Wolansky et al. 2006). In the present study, we used a mixture of these 11 pyrethroids to test the hypothesis of dose addition.

We also tested the hypothesis that kinetic differences between the 11 pyrethroids would result in less than dose addition if all compounds were administered at once. Toxicokinetic differences for individual pyrethroids result in variation in the time of peak effects of more than 4 hr for the 11 pyrethroids tested (Soderlund et al. 2002; Wolansky et al. 2006; Wolansky and Harrill 2008). To test this hypothesis, we used two alternative oral dosing protocols: simultaneous (SLT) administration of all chemicals with a 2-hr dose-to-test interval, and a sequential (SQT) protocol where the 11 compounds were administered either 1, 2, or 4 hr before testing, depending on each chemical’s time of peak effect. In addition, we conducted a time course study to determine the time of peak effect for the mixture when administered using the SLT protocol. The empirical mixture data for both the SLT and SQT exposure protocols were fit to a threshold dose–response curve and compared with the theoretical outcome predicted by the additivity model using the SCR method of analysis [modeling procedures described by Casey et al. (2004)].

## Materials and Methods

### Subjects

Male Long-Evans rats (Charles River Laboratories Inc., Wilmington, MA, USA) were obtained at 55–57 days of age and housed two per cage in standard polycarbonate hanging cages (45 cm × 24 cm × 20 cm) containing heat-sterilized pine shavings (Beta Chips; Northeastern Products, Inc., Warrensburg, NY, USA). All animals were given a 5- to 9-day acclimation period and were maintained on a 12:12-hr photoperiod (0600:1800 hours). Food (Purina 5001 Lab Chow; Ralston-Purina, St. Louis, MO, USA) and tap water were provided *ad libitum*. Tap water (city water; Durham, NC, USA) was filtered through sand and activated charcoal filters and then rechlorinated to 4–5 ppm Cl^−^ before use. Colony rooms were maintained at 22 ± 2°C and relative humidity at 55 ± 20%. The facility is approved by the American Association for Accreditation of Laboratory Animal Care. All animals were treated humanely and with regard to alleviation of suffering. All experimental protocols were approved in advance by the National Health and Environmental Effects Research Laboratory’s Animal Care and Use Committee.

### Chemicals

Technical grade samples of pyrethroids were kindly supplied by their manufacturers: permethrin, bifenthrin, and cypermethrin (FMC Corporation, Philadelphia, PA, USA); esfenvalerate (Dupont Crop Protection, Wilmington, DE, USA); deltamethrin and β-cyfluthrin (Bayer CropScience LP, Research Triangle Park, NC, USA); tefluthrin and λ-cyhalothrin (Syngenta Crop Protection, Greensboro, NC, USA); and fenpropathrin, resmethrin, and *S*-bioallethrin (Valent USA Corp., Walnut Creek, CA, USA). Information on the chemical purity and isomer composition was reported previously (Wolansky et al. 2006). Doses were calculated based on percent active ingredient in the technical product. Mixture stock and dosing solutions were prepared daily by codissolving pyrethroids in corn oil (Sigma Chemical Co., St. Louis, MO, USA) according to the dosing protocol described below. Solutions were stirred and gently heated (40–50°C) before dosing to assure full solubility and then used at room temperature.

### Mixture composition

The fixed ratio of individual pyrethroids in the stock solution (i.e., *a_i_* proportion; for each chemical *i*, *a_i_* = dose*_i_*/dose_mixture_) was based on the individual relative potency factors (RPFs) obtained from single-compound assays (Wolansky et al. 2006). Each RPF was calculated as the ratio of the ED_30_ for the index compound (i.e., ED_30_ for deltamethrin = 2.50 mg/kg) divided by the ED_30_ for each chemical. The ED_30_ was chosen as a biologically significant effect on motor activity (Crofton et al. 1991). The absolute doses of each chemical in the stock solution (i.e., the highest mixture dose examined) were equal to 33% of the ED_30_ for the chemical [see also Supplemental Material, Table 1 (doi:10.1289/ehp.0900667. S1 via http://dx.doi.org/)]. This dose is approximately 20% lower than the threshold dose previously calculated using the SCR approach on each individual dose–effect data set (Wolansky et al. 2006). Table 1 lists the chemical names, potency information (derived from Wolansky et al. 2006), mixing ratios, and stock mixture solution composition. Two sets of stock solutions were made. Stock A contained all 11 chemicals in the ratio and amounts described above. The second set, used for the sequential dosing, consisted of three separate stocks: Stock B1 contained *S*-bioallethrin only; stock B2 contained permethrin, cypermethrin, deltamethrin, esfenvalerate, β-cyfluthrin, fenpropathrin, tefluthrin, and λ-cyhalothrin; and stock B3 contained bifenthrin and resmethrin [see Supplemental Material, Table 2 (doi:10.1289/ehp.0900667. S1)]. The overall ratio and composition of stocks B1, B2, and B3 were equivalent to stock A. All dosing solutions were made daily.

### Exposure

Before dosing, animals were moved from the colony room to an isolated dosing room within the testing laboratory. After a 1-hr acclimation, animals were removed from home cages, dosed, and then returned to the home cages until the next dosing time or testing. All rats were randomly assigned to treatment groups. Body weights were counterbalanced across groups. Experimentally naive groups of rats were used for each experiment. Dose selection for the dose–response studies was based on pilot work (data not shown), with the goal to identify at least two no-effect levels.

We used vehicle control and two dose levels for the mixture time-course study: 0 (corn oil only), 76, and 152 mg pyrethroid/kg. The two doses were chosen to produce mild and moderate clinical signs of pyrethroid exposure. The high dose produced mild tremor in most of the animals that lasted from 1 to 4 hr, whereas the lower dose produced a small percentage of animals exhibiting transient burrowing and pawing behaviors but no tremor. Independent groups of animals were exposed for 1, 2, 4, 8, 24, or 48 hr before testing (*n*= 8 or 12 per group, except at 48 hr, *n* = 4 for the 76 mg/kg/group).

We used two experimental designs for the dose response that differed only in the dosing protocol used to administer all the chemicals. The SLT protocol used mixture stock A, whereby all 11 pyrethroids were administered at the same time in one mixture 2 hr before testing (three replicate test blocks with four rats per mixture dose per block). The 2-hr time point was based on the time of peak effect of the mixture determined in the time-course study. The SQT protocol used mixture stocks B1, B2, and B3 (two replicate test blocks with six rats per mixture dose per block). Because of known differences in the kinetics and time course of effects of the different pyrethroids, this protocol allowed sequential dosing where the 11 pyrethroids were administered according to their previously determined time of peak effects (Table 2). The time between dosing and testing for the three mixture stocks and dilutions were 1 hr for stock B1, 2 hr for stock B2, and 4 hr for stock B3. Seven mixture dosages, from 1% to 100% of the concentration of the stock solution, were tested in each dose–response experiment as follows: SLT protocol, 1%, 4%, 10%, 33%, 50%, 66%, and 100%; SQT protocol, 1%, 4%, 20%, 30%, 40%, 50%, and 100%.

### Motor activity testing

The same end point and testing procedures used in single-compound assessments (Wolansky et al. 2006) were used to examine mixture dose–effect relationships. Rats were transferred from the holding room to the adjacent testing room in individual polycarbonate transfer cages and were allowed to acclimate for 5 min before testing. Motor activity was then measured for 1 hr using 16 figure-eight mazes, each consisting of a series of interconnected alleys (10 × 10 cm) converging on a central arena and covered with transparent acrylic plastic (Norton et al. 1975; Reiter et al. 1975). Total activity was calculated as the sum of horizontal and vertical activity photocell counts. Photobeam calibration was checked daily before testing. Maze assignments, order of testing, and time of day were counterbalanced across treatment groups. All testing was conducted between 0900 and 1700 hours.

### Statistical analysis

We analyzed motor activity data (i.e., total photocell counts for the 1-hr test session) by two-way analysis of variance (ANOVA) using SAS software version 9.1 (SAS Institute Inc., Cary, NC, USA), with mixture dose and time as independent variables. For the SQT and SLT mixture-dose studies, a two-way ANOVA was used, with block and mixture-dose treatment as independent variables and total activity counts as the dependent variable. We performed mean contrast testing using Duncan’s new multiple range test (SAS). Data from one testing run in the time-course study (*n* = 8 rats) were excluded from formal analysis because of excessive noise from building construction. In addition, one rat was excluded because of excessive toxicity (8 hr, high dose). In the SLT experiment, data from one rat (1.5 mg/kg), and in the SQT experiment data from three rats (one each from the 1.5, 30.5, and 76.2 mg/kg groups), were excluded because of aspiration of the gavage fluid into the lungs.

Determination of departure from additivity for the motor activity data from two mixture dose–response studies (SLT and SQT protocols) used the SCR method. The definition of additivity given by Berenbaum (1985) can be related to the isobologram for a combination of chemicals (Loewe 1953; Loewe and Muischnek 1926) through the interaction index. That is, in a combination of *c* (here, *c* = 11) chemicals, *E_i_* represents the concentration or dose of the *i*th component alone that yields a fixed response (i.e., ED_30_), and *x_i_* represents the concentration/dose of the *i*th component in combination with the *c* agents that yield the same response. According to this definition, if the substances interact in an additive fashion, then


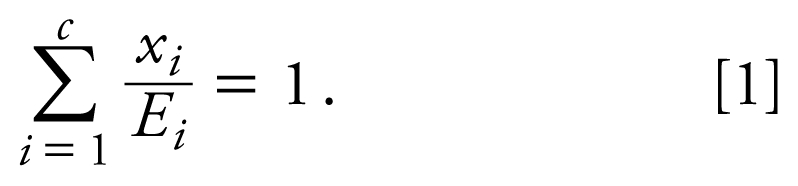


If the left-hand side of Equation 1, termed the “interaction index,” is < 1, then a greater than additive interaction (e.g., synergism) can be claimed at the combination of interest. If the left-hand side of Equation 1 is > 1, then a less than additive interaction (e.g., antagonism) can be claimed with the combination. This definition of additivity is a general form for dose addition. It should be pointed out that use of the toxic equivalence factor approach (Safe 1998) assumes common dose–response slopes across the chemicals under study; the general dose-addition definition of Equation 1 does not require such an assumption.

We combined the 11 chemicals according to the specified mixing ratio (Table 1) and evaluated them experimentally. The mixing ratio is denoted in terms of the proportion, *a_i_*, of each chemical in the mixture (Table 1) such that the summation of *a_i_* for the 11 chemicals equals 1, and the dose *x_i_* of each chemical in the mixture is


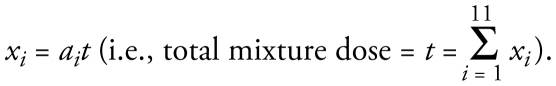


The SCR approach (Casey et al. 2004) allows for different slope parameters for each chemical and fixed-ratio mixture. The single-chemical data were modeled (termed the additivity model) using a nonlinear exponential threshold model for the mean motor activity (percent of control) of the form


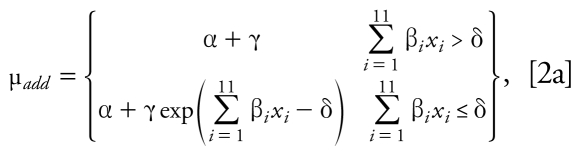


where α + γ = 100, α is the maximum effect parameter, *x_i_* is the absolute dose of the *i*th chemical, β*_i_* are the slope parameters for the individual chemicals (*i* = 1, . . . , 11), and δ is the threshold parameter such that the dose threshold for each individual chemical is given by δ*_i_* = δ/β*_i_*, *i* = 1, . . . , 11. The γ parameter was constrained to be γ = 100 – α, so that the mean response for the vehicle-control groups is 100%. It is important to note that the form of the additivity model does not include information about scheduling of dosing, because single-chemical data were available only with the SLT protocol. Therefore, for the present study we assumed that the model represented by Equation 2a is an additivity model for the case where the timing of the dosing has a negligible effect.

When the dose thresholds for all single chemicals are estimated outside of the experimental region, the model in Equation 2a is overparameterized. The corresponding nonlinear smooth additivity model is given by


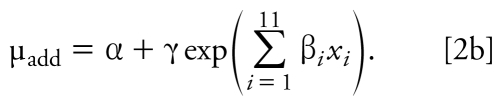


We estimated unknown parameters using the maximum quasi-likelihood method (McCullagh and Nelder 1989). The estimated additivity model was used to predict the mean response along the fixed mixing ratio of the 11 chemicals in terms of total dose. Threshold estimates, ED_30_ values, ED_10_ values (i.e., the response associated with a 10% motor activity decrease), and the corresponding large-sample 95% confidence intervals (CIs) for each single chemical were computed, as well as dose thresholds, ED_10_ values, and ED_30_ values for the mixture. In this work, all of these parameter estimates were computed using empirical data from single compounds and mixtures. However, for single chemicals, dose thresholds, ED_30_ values, and RPFs for motor function had been previously obtained using the same SCR model parameterized using only experimental data from single compounds (Wolansky et al. 2006). All 11 chemicals in this study are associated with significant decreases in motor activity as their doses increase, as evidenced by negative and significant slope parameters. The significance of the thresholds can be described by the significance of the threshold parameter (δ_add_) in the additivity model (*p* < 0.001) and the 95% CIs on the dose thresholds that did not include zero (Wolansky et al. 2006). We estimated the curve for the mixture dose–response relationship using the model expressed by


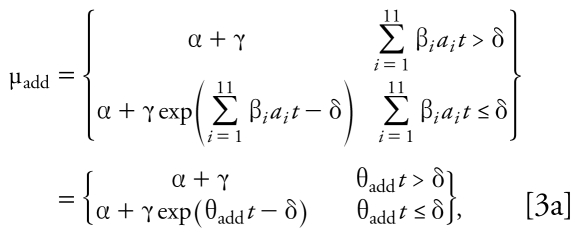


which is associated with the threshold additivity model given in Equation 2a, or


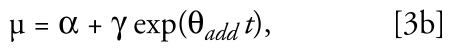


which is associated with the nonlinear additivity model given in Equation 2b.

Thus, the slope parameter associated with mixture along the specified fixed-ratio ray under additivity is


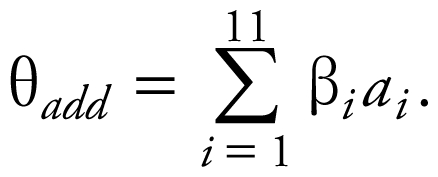


By replacing the unknown parameters in Equations 3a and 3b with parameter estimates, a plot of the dose–response curve under additivity for a specified fixed ratio of the chemicals was produced. The predicted mixture dose–response relationship was estimated using only single-chemical dose– response data (Equation 2) and then predicting along the mixture ray with the constraint of additivity given in Equation 1.

Following Gennings et al. (2002) and Casey et al. (2004), the mixture data along the fixed mixture ray is fit to a similarly parameterized mixture model of the form


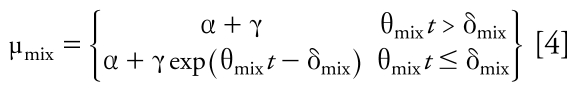


for a threshold model. The hypothesis of additivity along the specified ratio of the chemicals is a hypothesis of coincidence (i.e., the relationships are the same) between the additivity model in Equation 2 and the mixture model given in Equation 4 (i.e., for the threshold additivity models),


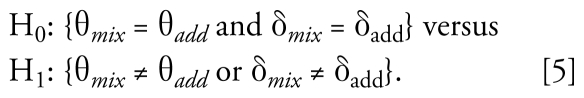


To determine whether there was a statistically significant deviation from additivity, we used a quasi-likelihood ratio test to compare the empirical mixture model with the restricted additivity model based on an *F*-distribution (e.g., Casey et al. 2004). The restricted additivity model (Casey et al. 2004) included only the single-chemical dose–response model parameters but used both the single-chemical and mixture data. We used this restricted model to predict the mean responses for the mixture data using the constraint of additivity given in Equation 1. Finally, we compared the predicted responses from the mixture data under the hypothesis of additivity (Casey et al. 2004; Gennings et al. 2002) with the observed sample means using an *F*-test.

## Results

We observed no mortality in the experiments in this study. The two higher mixture dosages (i.e., 76.2 and 152.4 mg/kg) evoked few clinical signs of pyrethroid toxicity. Mild whole-body tremors were present in most animals at the highest mixture dose (152.4 mg/kg) from 1 to 4 hr postdosing. Signs of high-dose pyrethroid toxicity such as excessive salivation, whole-body tremors, and choreoathetotic movements (Aldridge 1990; McDaniel and Moser 1993; Soderlund et al. 2002) were not observed in any animals, with one exception: One rat in the time-course experiment exhibited clinical signs of excessive pyrethroid exposure and raspy breath sounds, possibly due to partial aspiration of the gavage solution into the lungs. Data from this animal were not used.

**Figure 1:**
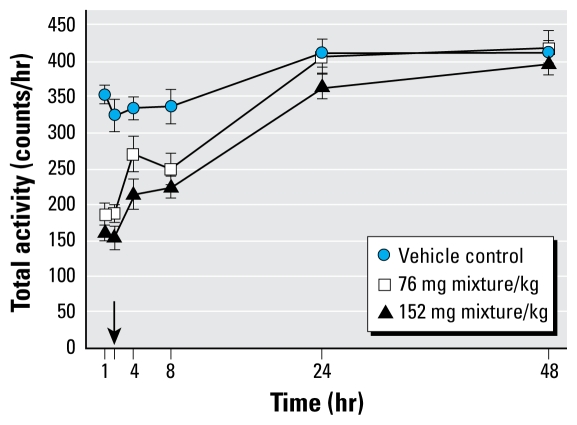
Time course of cumulative effects of 11 pyrethroids on figure-eight maze activity (mean ± SE). The arrow indicates the time of peak effects for the tested mixture.

The time-course study revealed a rapid decline in motor activity with a peak decrease at 1–2 hr postdosing (Figure 1). Activity recovered to control levels at 24–48 hr postdosing. The time course of effects was similar for both mixture-dose groups. These conclusions were supported by a significant mixture-dose × time interaction [*F*(10,147) = 5.18, *p* < 0.0001] and significant main effects of mixture dose [*F*(2,147) = 62.48, *p* < 0.0001] and testing time [*F*(4,147) = 15.5, *p* < 0.0001]. The activity of all mixture-dose groups was significantly decreased compared with controls at 1, 2, 4, and 8 hr (*p*< 0.05).

Parameter estimates and corresponding *p*-values from the additivity model in Equation 2a, from the simultaneously fit single-chemical data and mixture data, are provided in Table 3. The slope parameters associated with each of the 11 single chemicals (β values) and for the fixed-ratio mixtures (θ values) were negative and significant, indicating that as the dose of each individual chemical or the total dose of the mixture increases, the mean motor activity decreases.

**Figure 2:**
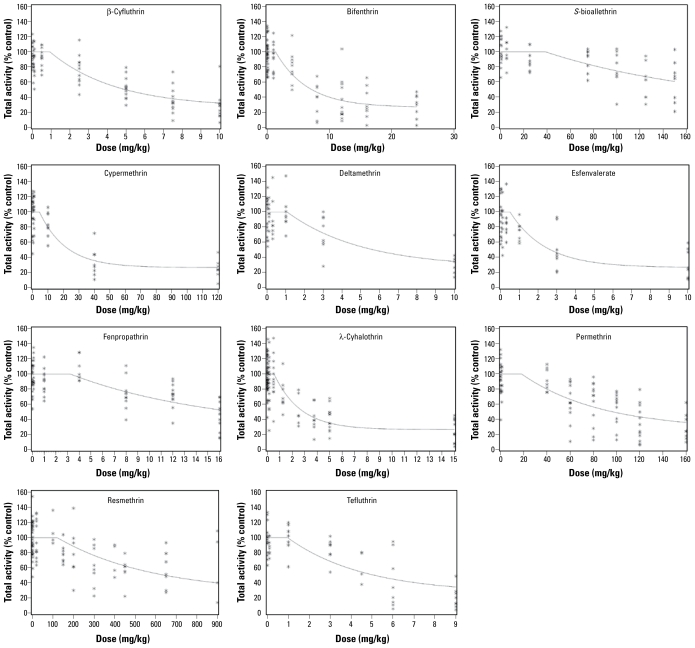
Observed data (individual data points) and the model-predicted dose–response curve from the additivity threshold model given in Equation 2a for each of the 11 pyrethroids.

Figure 2 shows the plots of individual data points for each chemical and the fit dose response using the additivity model. These plots illustrate the wide potency range of the individual chemicals, from 10 to 900 mg/kg, and the dose-related decrease in activity for all 11 chemicals. These plots also illustrate the estimated thresholds for each of the dose–response functions. Table 3 lists the estimated model parameters from the threshold additivity model and the mixture model fit simultaneously. The simultaneous fit of the additivity model and the mixture model accommodates a common maximum effect parameter (α), which was estimated to be 26.5 (i.e., 26.5% of control; Table 1). The γ parameter was constrained to be γ = 100 – α, so that the mean response for the control groups is 100%. Tables 4 and 5 list the model estimates for the threshold dose and ED_30_ dose. Note that the individual chemical estimates in Tables 3–5 are in some cases marginally different than previously published estimates (Wolansky et al. 2006) because of the inclusion of the mixtures data in the SCR model in the present study: The empirical mixtures data were not available for inclusion at the time the previous estimates were published.

**Figure 3:**
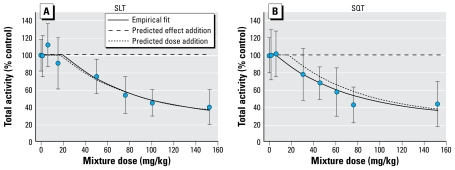
Dose–response relationships for the cumulative effects of 11 pyrethroids on figure-eight maze activity (mean ± SD). (*A*) SLT group. (*B*) SQT group. The departure of the experimental data from the predictive curve modeled assuming dose addition was not significant.

The mixture dose–response studies demonstrated dose-related decreases in activity regardless of the dosing protocol (Figure 3). For the SQT group, the pyrethroid mixture decreased activity by approximately 58% in the two highest mixture-dose groups, with significant decreases in all mixture doses ≥ 30.4 mg/kg (*p* < 0.05). There was no interaction between testing block and mixture dose [*F*(14,92) = 0.66, *p* < 0.8030], but there were significant main effects of dose [*F*(7,92) = 12.84, *p* < 0.0001] and block [*F*(2,92) = 6.15, *p* < 0.0035]. The main effect of block was due to slight differences in the overall baseline for activity counts over the different test days (data not shown). For the SLT protocol, the pyrethroid mixture decreased activity by 60% in the highest mixture-dose group, with significant decreases in all mixture doses ≥ 50.2 mg/kg (*p* < 0.05). There was no interaction between testing block and mixture dose [*F*(14,94) = 1.24, *p* < 0.2643], but there were significant main effects of dose [*F*(7,94) = 20.45, *p* < 0.0001] and block [*F*(2,94) = 2.85, *p* < 0.0414]. Because of the lack of interaction between replicate testing blocks and mixture dose, and the significant main effect of replicate block, we conducted all additional analyses on the motor activity counts expressed as percentage of block control values.

Results of the SCR method demonstrated no significant difference between the predicted response and the empirical data for both the SLT and SQT exposure protocols. Table 3 lists the slope estimates for individual chemicals and the dosing protocols, as well as the threshold estimates for both protocols. The empirical fit for the mixture administered using the SLT protocol was not different from that predicted assuming additivity, and the null hypothesis was not rejected [*F*(2,1037) = 0.015, *p* = 0.985; Figure 3A]. The small shift to the left in the dose–response relationship between the empirical and predicted curves for the SQT protocol (Figure 3B) was not significant, and the null hypothesis was not rejected [*F*(2,1037) = 2.65, *p* = 0.071]. The two thresholds were not statistically different; however, the SQT protocol threshold value (5.38 mg/kg) was 3.7-fold lower than that using the SLT protocol (19.82 mg/kg). This difference was 1.5-fold when we compared mixture ED_30_ values (SQT, 29.27 mg/kg, vs. SLT, 49.81 mg/kg). The CIs were wide and included zero, and although the threshold for the mixture administered using the SQT dosing protocol was numerically lower than that for the mixture where the chemicals were administered together at once (see Figure 3A,B), a test of coincidence in the two mixture curves was not rejected [*F*(2,1037)= 0.90, *p*= 0.407; Table 3].

## Discussion

In the present study we tested the hypothesis that the combined action of 11 pyrethroid insecticides on motor function is predicted by dose addition. We designed the mixture so that the highest mixture dose contained doses of each pyrethroid that were below their individual thresholds for effect. The results demonstrated that the additivity model predicted the measured effects on behavior in rats. The present results provide the first *in vivo* evidence on cumulative actions of pyrethroid mixtures in mammals. These data suggest that dose-additive approaches should be used when assessing the risk of exposures to chemical mixtures that contain pyrethroids.

The time course of effect of the pyrethroid mixture was consistent with patterns reported for a number of single-compound assessments. The time course for the mixture showed a maximum decrease in activity at 1–2 hr and recovery within 24–48 hr. Previous reports from studies using similar dosing protocols in rats demonstrated maximal decreases in figure-eight maze activity at 1–4 hr postdosing (Crofton et al. 1995; Crofton and Reiter 1984, 1988; McDaniel and Moser 1993; Wolansky et al. 2006). In addition, allethrin, *S*-bioallethrin, permethrin, fenvalerate, delta-methrin, and cypermethrin evoke alterations in motor-related end points in small rodents evident as early as 0.5–1.5 hr after systemic exposure (De Souza Spinosa et al. 1999; Hoy et al. 2000; McDaniel and Moser 1993; Nishimura et al. 1984; Wolansky et al. 2006). Likewise, the extended effect on activity through 8 hr is consistent with the prolonged syndromes evoked by resmethrin and bifenthrin (Crofton and Reiter 1984; Holton et al. 1997; Soderlund et al. 2002; White et al. 1976; Wolansky et al. 2006, 2007). Reports of longer times to onset after acute exposures (e.g., Soderlund et al. 2002) have been attributed to larger dosing volumes that delay absorption (Kim et al. 2007; Wolansky et al. 2007). The present data are also consistent with the fact that most of the 11 chemicals have individual times to peak effects of 1.5–2.5 hr postexposure (Table 2). These data support the hypothesis that the toxicokinetics of pyrethroids may not be altered in mixtures composed of low-levels of the individual insecticides. Toxicokinetic studies of pyrethroid mixtures are needed to test this hypothesis.

The mixture of 11 pyrethroids produced dose-dependent decreases in motor activity. This is consistent with previous reports of dose-related decreases in activity in the figure-eight maze in rats (Crofton et al. 1995; Crofton and Reiter 1984, 1988; Gilbert et al. 1990; McDaniel and Moser 1993; Reiter et al. 1981) and in other assessments with motor end points carried out in mice (Chanh et al. 1984; Mandhane and Chopde 1997). Thus, decreased locomotor behavior appears as a common finding of acute pyrethroid exposure to both individual compounds and mixtures.

An important finding of the present study is that low doses of the individual chemicals, when combined in a mixture, decreased motor activity. The threshold for decreased activity was 5.4 mg total pyrethroids/kg when the SQT protocol was used (Figure 3). The absolute amount of each individual pyrethroid at this mixture dose is approximately 3% of the threshold dose for altering motor activity when given alone in the single-compound assays (Table 1). These data clearly demonstrate two key findings. First, that effect addition, defined as a simple summation of the effects (i.e., sum of decreases in motor activity) of all chemicals in a mixture, underestimates the potency of the tested mixture by a wide margin. Second, these data demonstrate that low doses of individual pyrethroids, when acutely administered as a mixture, produce measurable effects on motor behavior in the rat.

We used the mixture experiments presented here to test two hypotheses. The first hypothesis, that the SCR threshold additivity model would predict the effects of an 11-pyrethroid mixture, was not rejected. There was no significant deviation between the predicted and empirical fits for data from either the SLT or SQT dosing protocols (Figure 3). Thus, dose addition can be used as a means to predict the effects of pyrethroid mixtures on motor activity. The second hypothesis, that kinetic differences between pyrethroids would lead to different effects if the individual pyrethroids were dosed according to the time of peak effect, was rejected. The model predicted the empirical effects of the 11-pyrethroid mixture for both the SLT and SQT protocols. These data are the first demonstration that dose addition correctly predicts the neurotoxic effects of a pyrethroid mixture composed of low-level, equitoxic doses of the individual chemicals.

The finding of dose addition for the 11 tested pyrethroids is consistent with a common target site, the voltage-gated sodium channel (Narahashi 2000; Soderlund et al. 2002). However, these results are not consistent with previous reports on exposures to multiple pyrethroids. Ray et al. (2006), using an *in vivo* hippocampal electrophysiologic model, demonstrated that deltamethrin and bioresmethrin did not act in an effect-additive or antagonistic manner. Burr and Ray (2004), using excised membrane patches from a neuroblastoma cell, showed that some combinations of type I and type II pyrethroids may compete for binding to a Cl^−^ channel target site. Furthermore, these authors reported that binary mixtures did not lead to effect-additive outcomes. Song and Narahashi (1996) concluded that tetramethrin may displace fenvalerate or interact allosterically with sodium-channel protein binding sites in an *ex vivo* rat dorsal root ganglion preparation. Although these reports suggest that pyrethroids do not act in an effect-additive manner, the experimental designs used preclude any definitive conclusions concerning additivity from these reports. First, the electrophysiologic work (Ray et al. 2006) used dose levels that exceeded known lethal doses. In addition, all of these previous reports lacked, either by study design or by statistical approach, the ability to test the hypothesis of additivity. The use of rigorous statistical models is critical for testing hypotheses of effect addition or dose addition and determining whether antagonism or synergism exists (Feron and Groten 2002; Gennings et al. 2004; Hertzberg and Teuschler 2002; LeBlanc and Olmstead 2004; Teuschler 2007).

The finding of dose addition for both the SLT and SQT protocols suggests a lack of toxicokinetic or enzymatic interactions at low doses, which has been shown for other mixtures (El-Masri et al. 1996a, 1996b, 2004). Alternatively, the present model may be unable to detect deviations from dose addition that might result from exposure to a complex mixture where most of the chemicals have similar time courses of effect. As shown in Table 2, 9 of the 11 pyrethroids had times of peak effect between 1 and 2.5 hr. The scarcity of toxicokinetic models for pyrethroids (Mirfazaelian et al. 2006) and the absence of any mixture models preclude any definitive conclusion on this issue. However, the present findings clearly indicate that small differences in the time of administration did not affect measured outcome (i.e., general motor function output in a maze).

The extrapolation of these findings to human exposures is currently tempered by a number of uncertainties. Humans are routinely exposed to multiple pyrethroids; however, concurrent exposures may be limited to only a small number of pyrethroids (Lu et al. 2009; Tulve et al. 2008), nowhere near the simultaneous exposure to 11 pyrethroids used here. In addition, the composition of the 11-chemical mixture was based on individual chemical potency (Table 1), not on environmental exposures. Whether other mixtures with a smaller number of chemicals and different chemical ratios will be dose additive is unknown.

Extrapolation of the present findings to humans is also hampered by an inability to compare exposures between species. The present work employed acute oral gavage exposures to rats. Human exposures from dietary and environmental residues are likely to be much lower compared with the rat acute oral exposures used in the present study. Acute oral bolus doses may result in higher peak tissue concentrations, compared with dietary and dermal human exposures (Conolly et al. 1999). Urinary levels of pyrethroid metabolites range from nondetectable to as high as 50 μg/L, with median levels between 1 and 5 μg/L (Lu et al. 2009; Morgan et al. 2007). Comparable data are not available in rats. Toxicokinetic models are needed that will allow comparison between effective doses in rats and aggregate human exposures.

## Conclusions

In summary, the present data demonstrate that subthreshold doses of individual pyrethroids, when combined in a mixture, produce measurable neurotoxicity in rats. These findings provide the first *in vivo* evidence of cumulative actions of pyrethroid mixtures in mammals and suggest that dose-additive approaches should be used for considering the combined toxicity of pyrethroid insecticides.

## Figures and Tables

**Table 1:** Summary of pyrethroid type, threshold dose, ED_30_, percentage of total mixture dose mass, and the absolute dose for each chemical.

Pyrethroid	Type	Threshold^a^ (mg/kg)	ED_30_*a* (mg/kg)	Percent total mixture dose	Pyrethroid dose (mg/kg)in stock mixture
β-Cyfluthrin	II	0.88	2.21	0.49	0.74
Bifenthrin	I	1.28	3.21	0.70	1.07
*S*-Bioallethrin	I	36.02	90.48	19.82	30.2
Cypermethrin	II	4.26	10.70	2.34	3.57
Deltamethrin	II	0.99	2.51	0.55	0.84
Esfenvalerate	II	0.48	1.20	0.26	0.40
Fenpropathrin	I/II	3.06	7.70	1.69	2.57
λ-Cyhalothrin	II	0.52	1.32	0.29	0.44
Permethrin	I	16.99	42.66	9.31	14.2
Resmethrin	I	116.60	292.80	64.10	97.6
Tefluthrin	I	0.90	2.26	0.49	0.75

The fixed mixing ratio was based on the ratio of the ED_30_ for the each chemical compared with an index compound (i.e., ED_30_ for deltamethrin = 2.50 mg/kg).

a Data from Wolansky et al. (2006).

**Table 2:** Individual times to peak effect and the mixture dosing protocols for each chemical.

Compound	Individual time to peak effect (hr)	Mixture dosing protocol (hours before testing)	
		SLT dosing	SQT dosing
*S*-Bioallethrin	1	2	1
Permethrin	1.5	2	1
Cypermethrin	1.5	2	2
Deltamethrin	2	2	2
Esfenvalerate	2	2	2
β-Cyfluthrin	2	2	2
Fenpropathrin	2	2	2
Tefluthrin	2	2	2
λ-Cyhalothrin	2.5	2	2
Bifenthrin	4	2	4
Resmethrin	4	2	4

**Table 3:** Estimated model parameters from the threshold additivity model (Equation 2a) and the mixture model (Equation 3a) fit simultaneously.

Parameter	Estimates	SE	*p*-Value
Single-chemical slope parameters			
α	26.49	2.14	< 0.001
β 1 (β-cyfluthrin)	−0.283	0.04	< 0.001
β 2 (bifenthrin)	−0.196	0.031	< 0.001
β 3 (*S*-bioallethrin)	−0.0069	0.0011	< 0.001
β 4 (cypermethrin)	−0.058	0.013	< 0.001
β 5 (deltamethrin)	−0.248	0.053	< 0.001
β 6 (esfenvalerate)	−0.529	0.105	< 0.001
β 7 (fenpropathrin)	−0.081	0.012	< 0.001
β 8 (λ-cyhalothrin)	−0.479	0.073	< 0.001
β 9 (permethrin)	−0.015	0.0018	< 0.001
β 10 (resmethrin)	−0.0022	0.0003	< 0.001
β 11 (tefluthrin)	−0.274	0.043	< 0.001
Mixture slope parameters			
θ_1_ (SLT)	−0.016	0.004	< 0.001
θ_2_ (SQT)	−0.015	0.003	< 0.001
Threshold parameters			
δ_add_	−0.262	0.077	< 0.001
δ_mix_1_ (SLT)	−0.307	0.303	0.31
δ_mix_2_ (SQT)	−0.08	0.102	0.433

Data are the estimated slopes for single chemicals (β parameters) and the mixture (θ parameters) administered using two alternative dosing protocols (i.e., SLT and SQT protocols), and the estimated thresholds (δ parameters) for the additivity model (δ_add_) and the two dosing protocols (δ_mix_1_ and δ_mix_2_).

**Table 4:** Estimated dose thresholds for each of the 11 chemicals with 95% large sample CIs.

Chemical	Threshold estimate^a^	SE	95% CI
β-Cyfluthrin	0.93	0.22	0.50 to 1.36
Bifenthrin	1.34	0.32	0.70 to 1.97
*S*-Bioallethrin	38.09	8.29	21.82 to 54.36
Cypermethrin	4.48	1.20	2.12 to 6.84
Deltamethrin	1.06	0.29	0.50 to 1.62
Esfenvalerate	0.50	0.13	0.25 to 0.75
Fenpropathrin	3.25	0.70	1.87 to 4.62
λ-Cyhalothrin	0.55	0.13	0.29 to 0.81
Permethrin	18.04	4.05	10.09 to 26.00
Resmethrin	121.64	25.68	71.25 to 172.03
Tefluthrin	0.96	0.22	0.52 to 1.40
Mixture 1 (SLT)	19.82	14.60	−8.83 to 48.47
Mixture 2 (SQT)	5.38	6.20	−6.78 to 17.54

a Threshold values for the individual chemicals vary slightly from those in Table 1 because of inclusion of the single-chemical and mixture data in the computation of the estimates (see “Materials and Methods” for details).

**Table 5:** Estimated ED_30_ values for each of the 11 chemicals with 95% large-sample CIs.

Chemical	ED_30_ estimate	SE	95% CI
β-Cyfluthrin	2.19	0.26	1.69 to 2.69
Bifenthrin	3.16	0.42	2.34 to 3.99
*S*-Bioallethrin	89.88	10.8	68.6 to 111.1
Cypermethrin	10.57	2.05	6.55 to 14.60
Deltamethrin	2.50	0.48	1.56 to 3.44
Esfenvalerate	1.17	0.20	0.78 to 1.57
Fenpropathrin	7.66	0.81	6.06 to 9.26
λ-Cyhalothrin	1.29	0.17	0.96 to 1.62
Permethrin	42.57	4.03	34.7 to 50.5
Resmethrin	287.03	30.7	226.7 to 347.4
Tefluthrin	2.26	0.29	1.70 to 2.83
Mixture 1 (SLT)	42.81	9.58	24.0 to 61.6
Mixture 2 (SQT)	29.27	4.52	20.4 to 38.1
